# Hypertrophic Cardiomyopathy—Past, Present and Future

**DOI:** 10.3390/jcm6120118

**Published:** 2017-12-12

**Authors:** Alphonsus C. Liew, Vassilios S. Vassiliou, Robert Cooper, Claire E. Raphael

**Affiliations:** 1 Royal Bournemouth Hospital, Bournemouth BH7 7DW, UK; Alphonsus.liew@hotmail.com; 2 Norwich Medical School, University of East Anglia; Norwich NR4 7UQ, UK; vass.nba@googlemail.com; 3 Norfolk and Norwich University Hospital, Norwich NR4 7UY, UK; 4 Royal Brompton and Imperial College, London SW3 6NP, UK; 5 Institute of Cardiovascular Medicine and Science, Liverpool Heart and Chest Hospital, Liverpool L14 3PE, UK; Rob.Cooper@lhch.nhs.uk; 6 Imperial College NHS Foundation Trust, London SW7 2AZ, UK; 7 Department of Cardiology, Imperial College NHS Foundation Trust, Hammersmith Hospital, Du Cane Road, London W12 0HS, UK

**Keywords:** hypertrophic cardiomyopathy, HCM, left ventricular outflow tract obstruction, LVOT

## Abstract

Hypertrophic cardiomyopathy (HCM) is the most common genetic cardiomyopathy with a prevalence of 1 in 500 in the general population. Since the first pathological case series at post mortem in 1957, we have come a long way in its understanding, diagnosis and management. Here, we will describe the history of our understanding of HCM including the initial disease findings, diagnostic methods and treatment options. We will review the current guidelines for the diagnosis and management of HCM, current gaps in the evidence base and discuss the new and promising developments in this field.

## 1. Introduction

In 1957, Robert Donald Teare, an English pathologist at St. George’s Hospital in London, reported the autopsy findings of eight patients with asymmetrical hypertrophy of the heart, seven of whom died suddenly [1]. The disease that affected these patients was later recognised as a distinct pathology, later named hypertrophic cardiomyopathy (HCM). With a phenotypic prevalence of 1 in 500 in the general population, HCM is the most common genetic cardiomyopathy [2]. It is characterised by abnormal thickening of the ventricular walls in the absence of abnormal loading conditions. It is the leading cause of sudden death in the young, with an incidence of sudden cardiac death (SCD) of 0.5–1% per year [3,4,5]. Patients with HCM are at increased risk of atrial fibrillation (AF), heart failure and myocardial ischaemia. In this review, we will outline the history of HCM and review current diagnostic modalities and management. We will discuss new and promising developments in the diagnosis of HCM and management of established disease. Potential therapeutic options for preclinical HCM are outside the scope of this review and will not be discussed.

## 2. Early Descriptions of HCM

Hypertrophic cardiomyopathy was first described by Vulpian, as “idiopathic hypertrophic subaortic stenosis” in 1868, after its anatomical anomaly [6]. This was shortly followed by similar descriptions in 1869 by Liouville and Hallopeau [7,8] in the Medical Gazette of Paris. Close to a century later in England, Brock reported three cases of LVOT (Left ventricular outflow tract) hypertrophy and suggested systemic hypertension to be the underlying cause [9]. A year later in 1958, Teare published a series of eight autopsy cases who had “asymmetrical hypertrophy or muscular hamartoma of the heart” [1]. All patients suffered a sudden death, with one exception, who died six hours post-mitral valvotomy. 

In the same year, Bercu et al. published their post-mortem findings of a patient who was diagnosed clinically as having aortic stenosis and was referred for aortic valvulotomy [10]. During the operation, the aortic valve cusps were found to be structurally normal and exploration of the left ventricle was not possible as it was “too small to admit the surgeon’s finger” [10]. The patient died shortly after surgery and post-mortem revealed marked myocardial hypertrophy in the absence of chamber dilatation. Due to the similarities in presentation and clinical findings to aortic stenosis, Bercu et al. dubbed it “pseudoaortic stenosis” [10]. 

By 1961, although still poorly understood, this disease entity was recognised to be distinct from aortic stenosis and termed idiopathic hypertrophic subaortic stenosis (IHSS). As two-dimensional echocardiography was not yet possible, diagnosis was made primarily during cardiac catheterisation. Since the aortic valve could not be directly visualised, distinguishing HCM from aortic stenosis remained challenging, even with invasive haemodynamics. Brockenbrough described the effect of post-premature ventricular contractions on aortic stenosis and HCM as a means of distinguishing the two conditions [11]. Frank–Starling law states that an increase in stretch of myocardial fibres results in an increase in myocardial contraction force, up to a certain point. Following a premature ventricular contraction (PVC), there is a compensatory pause before the next beat, thus leading to a longer diastolic filling time and a greater end diastolic volume (i.e., stretch). The greater myocardial stretch leads to greater contraction during systole.

**Figure 1:**
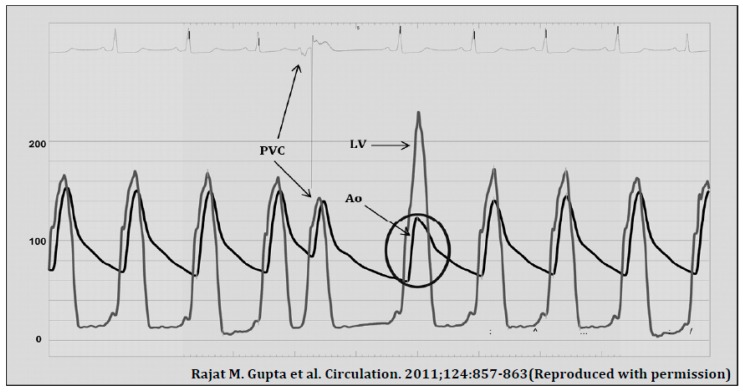
Brockenbrough sign after a premature ventricular contraction (PVC) in HCM (Hypertrophic cardiomyopathy). Pulse pressure, as recorded in the femoral artery, decreases in the post-extrasystolic beat (circled) while intracavity pressure in the LV (Left ventricle) increases due to greater obstruction in the LVOT (Left ventricular outflow tract). (Reproduced from [12] with permission).

In HCM, the obstruction in the LVOT is dynamic (orifice narrows during systole and relaxes during diastole), whereas, in aortic stenosis, the obstruction is constant. Brockenbrough et al. [11] hypothesised that a more forceful contraction, such as during the beat after a PVC, will narrow the orifice of the LVOT further, resulting in a narrower pulse pressure but a greater left ventricular pressure in HCM. In aortic stenosis, the obstruction is fixed and the LVOT is not affected by the greater contraction and therefore a physiological increase in pulse pressure will be observed with greater contraction. This became known as the Brockenbrough–Braunwald–Morrow sign (Figure 1). The higher gradient across the LVOT post-PVC will be associated with a louder murmur on auscultation. 

In 1963, the nonobstructive form of HCM was first described. Braunwald et al. found 14 patients, who, despite having left ventricular hypertrophy, brisk arterial pulses, a fourth heart sound and an ejection systolic murmur, did not have evidence of LVOTO (Left ventricular outflow tract obstruction) and were largely asymptomatic [13]. This was confirmed by subsequent studies [14,15,16,17]. Abbasi et al. in 1972 showed that M-Mode echocardiography may be used to diagnose nonobstructive HCM by demonstrating increased left ventricular wall thickness [18]. The timeline of events in the early development of HCM is summarised in Table 1. 

## 3. Left Ventricular Outflow Tract Obstruction (LVOTO)

Left ventricular outflow tract obstruction (LVOTO) is defined as a peak LVOT gradient of ≥30 mmHg. LVOTO has been shown to be associated with an increased risk of SCD [29,30,31,32] and progression to severe heart failure and death [33]. 

### Pathophysiology of LVOTO

**Figure 2:**
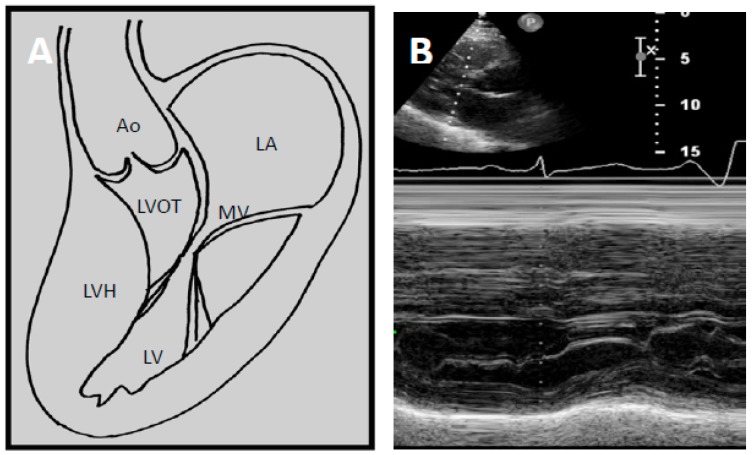

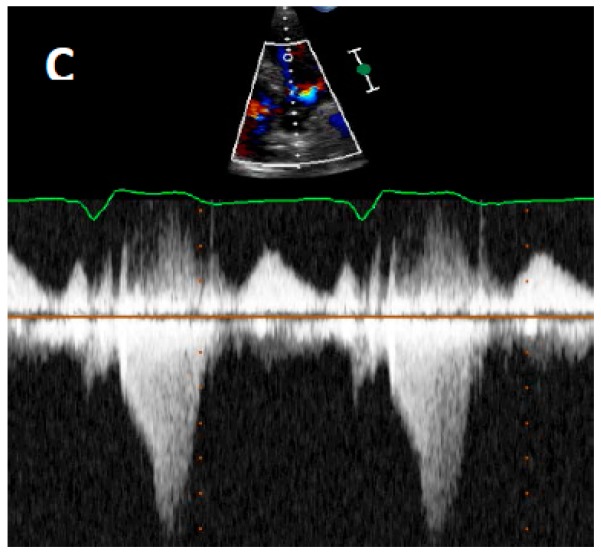
(**A**) Schematic demonstrating how left ventricular hypertrophy (LVH) with unfavourable mitral valve anatomy may result in obstruction of the left ventricular outflow tract. Echocardiography allows high quality imaging of the left ventricular outflow tract due to systolic anterior motion of the anterior mitral valve leaflet; (**B**) Abnormal motion of the mitral valve is well demonstrated on M-mode echocardiography. This may result in high velocities through the LVOT seen on continuous wave Doppler imaging as seen in (**C**). LA—Left atrium. MV—Mitral valve. LV—Left ventricle. LVOT—Left ventricular outflow tract, LVH—Left ventricular hypertrophy, Ao—Aort.

Two factors contribute to LVOTO; hypertrophy of the basal septum and systolic anterior motion (SAM) of the mitral leaflet. In HCM, the mitral valve architecture is abnormal, characterised by leaflet elongation, anterior displacement of the apparatus and papillary muscle abnormalities (Figure 2A). These characteristics make the mitral valve more susceptible to surrounding mechanical forces. As blood flows through the left ventricle in the presence of basal septal hypertrophy, it is directed posteriorly towards the mitral leaflets and then back anteriorly towards the LVOT [34,35]. When blood flows back towards the LVOT, it drags the mitral apparatus, resulting in an anterior motion of the leaflets. This SAM of the mitral leaflets is further reinforced by the Venturi effect produced by the higher velocity of blood flow induced by a narrower LVOT. As the mitral leaflets move anteriorly, they are directed towards the hypertrophied septum, touching the septum in severe instances. 

When the mitral valve touches the septum, it transiently but significantly narrows the LVOT, leading to a greater obstruction and a greater pressure difference across the LVOT. This pressure difference further drags the mitral leaflets against the septum, narrowing the orifice further, resulting in a reinforcing feedback loop that persists until the end of systole. This significantly reduces cardiac output and contributes to coronary flow abnormalities [36]. Where coaptation of the mitral leaflets is incomplete due to vigorous SAM, mitral regurgitation occurs. In mitral regurgitation secondary to SAM, the regurgitant jet is directed posteriorly, consistent with the angle of the leaflets. Regurgitant jets that are directed anteriorly or centrally suggest a different culprit.

## 4. Diagnosis

### 4.1. Cardiac Catheterisation

In its nascent days, the diagnosis of HCM revolved mainly around identifying dynamic LVOTO, a characteristic we now know only exists in approximately one-third of patients at rest. This was initially done via cardiac catheterization, which allowed pressure gradients across the LVOT to be measured. Although a reliable diagnostic tool, it was invasive, time-consuming, caused discomfort and was not without radiation and procedural risks.

### 4.2. Echocardiography

Echocardiography has transformed diagnostic testing in cardiology. In 1969, Moreyra et al. pioneered the use of echocardiography in diagnosing HCM [21]. The idea of SAM of the mitral leaflet in systole contributing or causing LVOT obstruction was first postulated by Bjork in 1965 at a symposium in London [20]. Due to the lack of supporting evidence at the time, this view was initially unpopular. However, this changed as subsequent angiographic contrast studies demonstrated a characteristic SAM of the mitral leaflet in systole leading to LVOT obstruction [37,38,39]. In 1969, Shah et al. confirmed the finding of SAM of the mitral leaflet using echocardiography and found it to be distinctive of HCM [22] (Figure 2B). The introduction of 2D echocardiography in 1972 allowed direct visualisation of the aortic valve and LVOT, allowing the non-invasive distinction of HCM and aortic stenosis [23]. 

## 5. Current Diagnostic Techniques

Two-dimensional (2D) echocardiography has long been the mainstay initial modality of choice in HCM diagnosis. It is used to outline cardiac structures and assess valvular and cardiac function. Doppler echocardiography at rest and during Valsalva manoeuvre is recommended to detect evidence of resting or latent LVOTO (Figure 2C). Left ventricular ejection fracture (LVEF) is usually normal or supra-normal in HCM, while the internal cavity left ventricular (LV) systolic dimensions are often small. As such, myocardial strain may be a better measure of systolic function than LVEF in HCM. Myocardial strain may be measured with Doppler myocardial strain imaging and 2D speckle-tracking technology [40]. Indeed, reduced LV myocardial strain in HCM has been shown to be associated with NSVT, appropriate ICD discharges and cardiac death [41,42,43]. Echocardiography provides detailed assessment of diastolic function, which is often abnormal in HCM. 

Today, the diagnosis of HCM relies on the identification of increased left ventricular wall thickness on imaging. This is most commonly via echocardiography. The European Society of Cardiology/American College of Cardiology Foundation (ESC/ACCF) diagnostic criteria for HCM is a wall thickness of ≥15 mm in at least one LV myocardial segment, in the absence of abnormal loading conditions such as hypertension and valvular disease [44,45]. A lower threshold of LV wall thickness ≥13 mm in at least one LV myocardial segment may be used in the presence of a first-degree relative with confirmed HCM [44,45]. HCM-phenocopies may be caused by Fabry’s disease, light chain amyloidosis, and drugs (e.g., steroids, tacrolimus). Recently, a link has been postulated between Hepatitis C virus infection and cardiomyopathy [46,47]. Further research in this area is needed.

### 5.1. Cardiovascular Magnetic Resonance (CMR)

**Figure 3:**
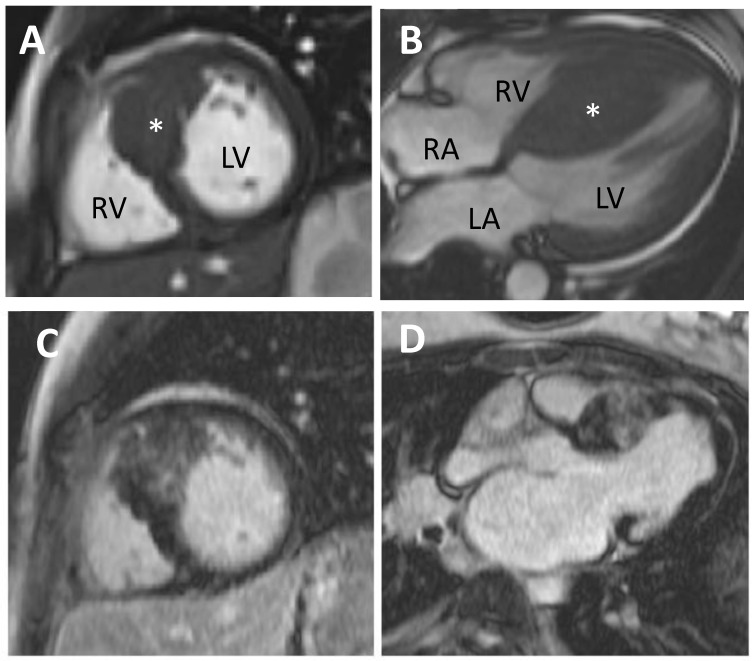
Hypertrophic cardiomyopathy imaged using cardiovascular magnetic resonance (CMR). (**A**) Short axis through the heart and asymmetrical left ventricular hypertrophy (starred); (**B**) Four chamber view of the heart; (**C**) Late gadolinium imaging showing patchy fibrosis of the hypertrophied area; (**D**) Same heart in the long axis. LV—Left ventricle, RV—Right ventricle, LA—Left atrium, RA—Right atrium.

CMR is a helpful diagnostic and risk stratification tool in HCM. It possesses excellent spatial and temporal resolution, allowing accurate assessment of ventricular volumes and anatomical structures (Figure 3). Unlike echocardiography, it is not restricted by poor acoustic windows and is more sensitive in detecting apical or focal HCM. Tissue characterisation with late gadolinium enhancement (LGE) allows detection and quantification of myocardial fibrosis (Figure 3C,D). Fibrosis is present in up to two-thirds of HCM patients [48,49,50]. Gadolinium contrast will accumulate in areas of myocardial fibrosis and is seen as bright tissue on inversion recovery sequences. The presence of fibrosis as assessed via LGE has been found to be a strong independent predictor of SCD, heart failure death, cardiac mortality and all-cause mortality in HCM, making CMR a useful prognostic imaging modality [51,52]. The extent of fibrosis as detected by LGE has also been suggested to be important when characterising risk of adverse outcomes [52]. However, technical variability in LGE quantification exists and is determined by the sequences used, the thresholds set for LGE identification and timing of post-contrast imaging [47]. Until this is standardised, formulating guidelines to aid risk stratification in HCM using LGE will remain a challenge. 

### 5.2. Positron Emission Tomography (PET)

PET is the gold-standard imaging modality for the non-invasive detection of ischaemia and is particularly valuable in identifying microvascular dysfunction through perfusion abnormalities. In the absence of epicardial coronary disease, coronary flow resistance is predominantly determined by the coronary microvasculature. In patients with normal or minimal coronary artery disease, a blunted increase in myocardial blood flow during cardiac stress may be present and is a strong independent predictor of heart failure, SCD, LV remodelling and cardiovascular mortality in HCM. Although perfusion abnormalities predict adverse outcomes, there is a lack of targeted treatment options for these abnormalities and so PET is not recommended for risk stratification [44,45].

### 5.3. Genetics

The first pathogenic mutation implicated in HCM was discovered by Geisterfer-Lowrance et al. in 1990 [25]. They found a missense mutation of the sarcomeric gene, β cardiac myosin heavy chain (βMHC), to be present in all members of two unrelated families with HCM and deduced that mutations in MHC genes were responsible for HCM. This led to a search for other pathogenic mutations in HCM and the discovery of several sarcomeric culprits including cardiac myosin-binding protein C (MYBPC3), cardiac muscle troponin T (TNNT2), cardiac troponin I (TNNI3), regulatory myosin light chain (MYL2), essential myosin light chain,α tropomyosin, and α cardiac actin in decreasing order of prevalence.

To date, nine genes have been definitively implicated in HCM [47,53,54,55,56,57] (Table 2). Up to 60% of patients who meet the HCM diagnostic criteria demonstrate a pathogenic sarcomeric mutation on genetic testing [58,59]. Genetic testing is recommended in patients who meet the diagnostic criteria for HCM to allow cascade screening in patients with a pathogenic mutation. Where a pathogenic mutation is identified, it aids the identification and monitoring of affected relatives [44,45]. Testing allows asymptomatic members of the family with the pathogenic mutation to be identified early and followed up regularly. When no pathogenic mutation is found in a relative of an affected genotype positive individual, they may be safely discharged from the clinic with advice to return for re-assessment if HCM-suggestive symptoms develop [44]. For HCM patients with no pathogenic mutation, clinical follow-up of first-degree relatives is typically clinical examination, ECG and transthoracic echocardiography on a regular basis.

## 6. Management of HCM

Historically, management of HCM has focussed on symptom relief and reducing the risk of fatal arrhythmic events. 

## 7. Sudden Cardiac Death-Risk Stratification and ICD Therapies

Prevention of SCD in HCM is complex. Various risk stratification schemes have been proposed. Risk stratification helps guide clinicians as to whether implantable cardioverter defibrillator (ICD) implantation should be recommended. The ICD detects sustained ventricular arrhythmias (ventricular fibrillation or ventricular tachycardia) and either attempts to use anti-tachycardia pacing to terminate ventricular tachycardia or delivers a cardioversion (“shock”).

ICD implantation will effectively treat VT or VF in HCM. However, determining an appropriate and acceptable threshold for ICD implantation is complicated. ICDs can adversely affect patients’ quality of life and psychological wellbeing through inappropriate discharges and driving restrictions, and have periprocedural risks such as infection, pneumothorax, pericardial effusion, lead displacement and death. 

The first ICD use in humans was pioneered by Mirowski et al. in 1980 [24]. An HCM patient was among the first individuals who received an ICD. Studies into the efficacy of ICD in SCD prevention in the HCM cohort were lacking until 2000. Maron et al. studied the use of ICD in 128 HCM patients with high SCD risk and found it to be effective in terminating fatal arrhythmias with an appropriate ICD discharge rate of 7% per year [27]. In 2002, the ACC/AHA/NASPE guidelines recommended (class IIb) the use of ICD in primary prevention of SCD in HCM [28].

Currently, two main models guiding ICD implantation are advocated by the ESC and American College of Cardiology Foundation/American Heart Association (ACCF/AHA), respectively [44,45]. The indication for ICD implantation from both models is clear-cut for patients who have survived a cardiac arrest from ventricular fibrillation (VF) or experienced sustained ventricular tachycardia (VT). The first model (HCM Risk-SCD), advocated by the ESC, is based on a large multicentre, retrospective, cohort study of 3675 patients. This study found seven risk factors that were independent predictors of SCD. These were:

- Maximal LV wall thickness,
- Left atrial diameter,
- Maximal LVOT gradient (at rest or by Valsalva),
- Family history of SCD,
- Unexplained syncope,
- Non-sustained ventricular tachycardia (NSVT), and
- Age.

Using these risk factors, they produced a formula predicting 5-year SCD risks with a discriminatory ability (C-statistic 0.7) that is comparable to the widely used CHAD_2_S_2_-VASc model used for estimating stroke risk in AF. Using the risk scores from this model, the ESC states that ICD implantation in patients with a 5-year SCD risk of; ≥6% should be considered, ≥4% to <6% may be considered, and <4% to be considered only in the presence of clinical features proven to have prognostic importance. All patients receiving ICD should have a life expectancy of >1 year. 

The second model, advocated by the ACCF/AHA, states that ICD implantation can be considered in the presence of ≥1 of the following risk factors:

- NSVT,
- family history of SCD,
- maximal LV wall thickness ≥30 mm,
- unexplained syncope, and
- abnormal blood pressure response during exercise (defined as a decrease in blood pressure or inability to increase blood pressure by ≥20 mmHg during exercise).

The implantation rates of both models differ quite significantly. Use of ACCF/AHA model resulted in an ICD implantation rate of 40–60% [5,60] of HCM patients compared to only 20–26% [60,61] when using the ESC model. Patients who received an ICD based on the ACCF/AHA model had a low annual rate of appropriate ICD discharges of approximately 2% [5,62] while the ESC model has been found to be relatively specific but with limited sensitivity in predicting SCD or appropriate ICD discharges [61]. The true percentage of patients who would benefit from ICD implantation is probably somewhere in between the two figures and the decision for ICD implantation should be guided by a clinician’s assessment of their individual patient. 

## 8. Management of LVOTO

### 8.1. Non-Invasive Therapy

Patients with LVOTO are advised to avoid factors that reduce the end diastolic volume (e.g., Dehydration, nitrates, phosphodiesterase inhibitors and dihydropyridine calcium channel blockers) or increase myocardial contractility and hence the degree of outflow tract obstruction (e.g., Inotropic drugs). Simple lifestyle measures such as weight loss and staying well hydrated are appropriate. Medical therapy comprises largely of negatively inotropic drugs and is aimed at reducing left ventricular wall contraction, reducing the narrowing of the outflow tract. Both ESC and ACCF/AHA have similar recommendations [44,45]: Non-vasodilating beta blockers e.g., bisoprolol are the first-line recommendation to improve symptoms in patients with LVOTO at rest or on provocation. Non-dihydropyridine calcium channel blockers (e.g., verapamil or diltiazem) are second line, while disopyramide is a useful adjunct to either beta blockers or calcium channel blockers. Some physicians choose to avoid the use of ACEi and ARB in HCM, particularly in LVOT obstruction where decrease in the pre-load may lead to worsening symptoms. However, a recent randomised, double-blind, placebo-controlled trial, INHERIT, which investigated the effect of losartan on left ventricular hypertrophy and fibrosis found ARBs to have a good safety profile in HCM patients with or without LVOT obstruction [63].

### 8.2. Invasive Therapy

Invasive therapy aims at reducing interventricular septum thickness and contractility, thus reducing LVOT gradient. Septal reduction therapy should be considered in symptomatic patients (NYHA III-IV) with a resting or maximum provoked LVOT gradient of ≥50 mmHg, despite optimal medical therapy [44,45]. It should also be considered in patients with recurrent exertional syncope secondary to LVOTO gradient that is ≥50 mmHg at rest or on provocation. There are currently two main forms of septal reduction; surgical myectomy and alcohol septal ablation (ASA).

Surgical myectomy involves the removal of a small amount (5–10 g) of hypertrophied tissue from the basal interventricular septum. Many centres now also incorporate an extended myectomy down to the level of the mid-septum. Morrow performed the first surgical myectomy in 1964 [19] in a cohort of highly symptomatic HCM patients (NYHA class III) with LVOT obstruction. Postoperatively, almost three-quarters of the patients who were followed up were asymptomatic (NYHA I) and the remaining were NYHA II. This became known as the Morrow procedure and was later modified over the years to involve resection of a greater area of myocardium.

Surgical myectomy has been the gold standard for septal reduction since its introduction in 1964. It obliterates or significantly reduces LVOT gradient in 90% of patients when performed in expert centres [64,65,66,67,68]. Since it has more long-term data on the younger population (children to young adults), it is the therapy of choice for this subgroup. Concomitant papillary muscle abnormalities, moderate-to-severe mitral regurgitation and/or significant mitral leaflet elongation may also be treated surgically at the time of myectomy [44,69]. 

Three decades after the first myectomy in HCM, Sigwart performed the first ever non-surgical septal reduction [26] using surgical alcohol. Alcohol septal ablation is a catheter-based technique that involves the injection of a small amount of desiccated alcohol (1.5–2.5 mL) into a septal perforator to induce myocardial necrosis and reduction in septal tissue. It has comparable results to surgical myectomy, demonstrating improvement in symptoms, functional status and LVOT gradient reduction [70]. Atrioventricular (AV) block is the most common complication of ASA (7–20%). Technical difficulty arises when the septal blood supply is ambiguous, making identification of the appropriate septal branch for alcohol injection complicated. Echocardiography with myocardial contrast is extremely helpful in this situation and may be used to confirm localisation of contrast to the basal septum prior to injection of alcohol. 

Both surgical myectomy and ASA may provide substantial symptomatic improvement, increased exercise capacity and reduction or obliteration of an LVOT gradient [71,72,73,74]. Reduction or obliteration of LVOTO has been associated with a relative risk reduction in SCD [75,76,77]. Post-operative survival following both procedures is comparable to that of the age- and sex- matched general population [68,78,79]. Ommen et al. compared survival between surgical treatment and medical management alone in patients with symptomatic obstructive HCM and demonstrated significantly lower all-cause mortality, HCM-related mortality and fatal arrhythmic events in the surgical group [68]. Although survival between ASA and medically managed groups has not been directly compared, patients who underwent ASA have been demonstrated to have similar long-term survival to patients with myectomy, implying an indirect benefit over medically treated patients with symptomatic obstructive HCM [71,74]. It is worth noting that the main indication for septal reduction therapy in HCM is to provide symptomatic relief in NYHA III-IV patients who are already on optimal medical therapy. In this regard, septal reduction therapy provides excellent long-term symptomatic benefits and SCD risk reduction [71,72,73,74,75,76,77]. Procedure-related mortality at experienced centres for both surgical myectomy and ASA is about 1% [68,78,80,81]. 

Septal reduction therapy decisions should be jointly made by a multidisciplinary team of expert cardiologists, cardiothoracic surgeon, a heart care team and the patient. Septal reduction therapies should be performed at dedicated HCM centres, as this is associated with lower rates of complications. 

## 9. Atrial Fibrillation

Atrial fibrillation has a prevalence of over 20% in the HCM population [82] compared to 1–2% in the general population [83]. This is likely due to raised left ventricular end diastolic pressure (LVEDP) related to ventricular dysfunction caused by a stiff hypertrophied left ventricle and/or LVOT obstruction. This leads to chronically elevated LA pressure, LA dilatation and remodelling. Atrial myopathy may also have a role in AF, as cardiomyocytes in HCM have been found to be abnormal not only in the ventricles but throughout the heart in HCM.

Thromboembolism is a common consequence of atrial fibrillation, with an estimated prevalence of up to 30% in the HCM population with AF [82]. It is often associated with clinical deterioration due to cardiac failure [84,85,86]. To date, the risk factors that have been found to predict AF in HCM include left atrial dimensions, the extent of septal hypertrophy, age and female sex [82]. 

### 9.1. Non-Invasive

Non-invasive management of AF in HCM focuses on rate control, thromboprophylaxis and restoration of sinus rhythm. Rate control is achieved with beta blockers or non-dihydropyridine calcium channel antagonists. Oral anticoagulation should be started in all HCM patients with AF where possible. Warfarin remains the anticoagulant recommended by the ESC/ACCF [44,45]. However, with growing long-term data, direct oral anticoagulants (DOACs) are gradually becoming more popular as they require less monitoring. CHA_2_DS_2_VASc score should not be used in risk stratification of HCM with AF as the risk of stroke is already high. Restoration of sinus rhythm should be considered in new onset AF after at least three weeks of sufficient anticoagulation or exclusion of left atrial appendage thrombus. 

### 9.2. Invasive

AF ablation may be considered as a method to restore sinus rhythm. There are limited reports of AF ablation outcomes in HCM, although procedural success rate seems to be lower in the HCM population compared to the general population (51.8% vs. 71.2% free from AF, respectively, at 1.8 years after more than one AF ablation and with greater persistent use of antiarrhythmics) [87]. However, AF is often associated with heart failure and/or deterioration in HCM, so the potential benefits from successful ablation are greater. 

## 10. Myocardial Ischaemia in HCM: Why Does It Happen and How Should We Manage It?

Ischaemia is a common finding in HCM. Up to half [88,89,90] of HCM patients experience chest pain, commonly occurring at rest [91]. Evidence of ischaemia in HCM has been found in post-mortem studies in the form of necrosis, fibrosis and neutrophilic infiltrates, usually in the absence of epicardial coronary artery disease. Abnormalities of the coronary microvasculature such as pathologic proliferation of the tunica intima and/or media, resulting in a small cross-sectional luminal area being seen in the post mortem study [92,93,94,95,96,97]. 

The impaired relaxation of hypertrophied and fibrotic ventricles during diastole will also lead to an increased intramural end diastolic pressure, while contraction of the hypertrophied ventricle during systole will result in greater compression of the intramyocardial vessels and reduced myocardial blood flow. LVOTO may further impair myocardial blood flow during systole [36]. 

While the presence of ischaemia is well-recognised in HCM, its effects on outcomes were largely understudied until 2003 when Cecchi et al. showed that extent of microvascular dysfunction measured using PET strongly predicted clinical deterioration and death in HCM [98]. Patients with marked perfusion abnormalities on PET had a ten-fold risk of death and twenty-fold risk of heart failure compared with patients with normal or near-normal perfusion. These patients were also at a higher risk of LV remodelling, systolic dysfunction and SCD [98,99,100,101,102]. However, it is important to note that this study preceded use of CMR and fibrosis and the additive data from perfusion imaging is not yet known. 

Guidelines for the management of microvascular dysfunction are largely absent. Data on the beneficial effects of treating perfusion abnormalities on long-term outcomes such as heart failure, mortality and SCD are currently limited. Therapies that reduce myocardial contractility (thereby reducing myocardial oxygen demand and LVOTO) and prolong diastole, such as beta blockers and calcium channel blockers, are probably beneficial. 

## 11. Future Directions in HCM

### 11.1. Diagnosis

#### 11.1.1. CMR

LGE in CMR is an excellent tool in identifying myocardial fibrosis. However, the LGE technology is based on a nulling method, which means it requires normal surrounding myocardium to detect areas of fibrosis. This constitutes a challenge in patients who have diffuse interstitial fibrosis. T1 mapping in CMR may provide a solution [103]. Recent studies have demonstrated that using T1 native and post-contrast values myocardium with diffuse fibrosis may be reliably distinguished from normal myocardium [104,105]. 

Diffusion tensor CMR (DT-CMR) studies the diffusion of water across microstructures. This is based on the concept that water molecules diffuse at different rates along microstructures of different anisotropy and orientation. Water molecules diffuse more easily along the direction that the microstructures are aligned in, and diffuse slower when going in a direction perpendicular to the alignment. Using DT-CMR, recent work has shed some light on the behaviour and orientation of cardiomyocyte sheetlets (the secondary organisation of cardiomyocytes) in HCM during systole and diastole [106]. In normal hearts, cardiomyocyte sheetlets evolve from a more wall-parallel orientation in diastole to a more wall-perpendicular orientation in systole, culminating in radial wall thickening. In HCM, however, the sheetlets were found to be in a wall-perpendicular orientation during diastole, demonstrating impaired diastolic relaxation. Further developments in DT-CMR may allow us to better understand the contractile mechanisms, which lead to diastolic impairment in HCM and guide its management. 

#### 11.1.2. Biomarkers

Myocardial fibrosis has long been a hallmark of HCM and has been assumed to be a result of repetitive ischaemic injury and consequent remodelling of LV tissue. A recent biomarker study looking at the C-terminal propeptide of type I procollagen (PICP), a by-product of type I collagen synthesis, have found that this biomarker was significantly raised in sarcomeric mutation carriers compared to their mutation-negative counterparts [107]. PICP was raised in all mutation-positive patients, with or without overt hypertrophy. However, the PICP: CITP ratio (an index of dynamic equilibrium between synthesis and degradation of type I collagen) was raised only in patients with hypertrophy. This suggests two things:

(1) Fibrotic development occurs when the equilibrium between collagen synthesis and degradation is disrupted
(2) Sarcomeric mutations may directly induce the development of fibrosis, irrespective of the presence of ischaemia.

If confirmed by subsequent studies, this may allow clinicians to identify HCM patients with high levels of PICP who may be at elevated risk of aggressive disease progression before the development of overt hypertrophy. 

#### 11.1.3. Genetics

Currently, genetic testing has little role to play in risk stratification in HCM and is primarily used for family screening. However, patients with multiple sarcomeric gene mutations have an increased risk of cardiovascular death, SCD and end-stage progression [108,109]. Although double-mutation carriers are rare, future large genetic cohort studies may define whether these patients would benefit from an ICD for primary prevention in the absence of conventional risk factors.

#### 11.1.4. Medical Therapy

Spironolactone has been demonstrated in an animal study to have an incremental effect in reducing cardiac hypertrophy when combined with captopril in rat models with HCM [110]. Its efficacy in producing similar effects in the human model remains to be explored.

Ranolazine has shown promising results in improving angina, heart failure symptoms, and quality of life in symptomatic HCM patients in a proof-of-concept study [111]. In HCM, there is a higher proportion of inward late sodium currents (I_NaL_) in cardiomyocytes. This prolongs the action potential and increases intracellular Ca^2+^ accumulation, increasing myocyte contraction, and thus greater energy expenditure and ischaemia. By inhibiting late sodium currents, Ranolazine is thought to reduce myocardial energy expenditure and potentially slow disease progression in HCM. The latter was shown to be possible in mice models but is yet to be demonstrated in human models [112].

Angiotensin receptor blockers (ARBs) have been postulated to be effective at inducing phenotypic regression in transgenic HCM [113,114,115,116]. Evidence for the efficacy of ARBs in the clinical setting has, however, been scarce. A rare and laudable double-blinded, placebo-controlled study, INHERIT, investigated the effects of losartan on LV mass reduction in HCM patients [63]. They compared the effects of losartan versus placebo in 133 HCM patients (64 in Losartan arm and 69 in placebo arm) in the reduction of LV mass and found no significant difference between the two, demonstrating that the beneficial effects of losartan may not be seen in humans. 

#### 11.1.5. Interventional

Endocardial radiofrequency ablation (ERA) is a relatively new interventional technique that offers an alternative to ASA in the treatment of LVOTO. It involves the application of radiofrequency lesions to the hypertrophied septum, inducing necrosis and the development of fibrosis within the septum. Although the effects on septal size are limited, the damaged myocardium does not contract and systolic excursion of the basal septum into the LVOT is reduced, reducing LVOTO [117,118]. ERA is a particularly attractive option for patients who do not want or are not fit for surgery but have unsuitable septal branch anatomy for ASA. It is, however, in its early stages with less than 100 procedures reported to date and more work is needed to validate its long-term efficacy.

#### 11.1.6. Molecular Therapy

MYK-461 is a novel molecular inhibitor of sarcomeric contraction. Discovered recently in a screen for direct inhibitors of the sarcomere [119], MYK-461 selectively inhibits myosin ATPase in mice, slowing the rate of phosphate release, the rate-limiting step in the chemomechanical cycle responsible for sacomeric force generation. The end result is reduced sarcomeric contractility without affecting chronotropy. Another study using MYK-461 in cats demonstrated a dose-dependent reduction in LVOTO and contractility, as well as elimination of SAM [120]. If demonstrated to be safe and effective in humans, MYK-461 may be an alternative to beta-blockers and calcium channel blockers as a drug that specifically targets the source of abnormality, the sarcomere. Rectifying the defect caused by sarcomeric mutations could change the pathophysiological course of HCM and prevent the development of its sequelae such as hypertrophy, myofibrillar disarray and fibrosis. 

## 12. Conclusions

Our understanding and management of HCM has come a long way since its first modern description in the 1950s. Management of HCM is complex and involves risk assessment of SCD and the treatment of complications resulting from the disease process, such as LVOTO, AF, ischaemia and heart failure. The developing role of genetic and molecular therapy to selectively target the defects of the sarcomere directly is particularly exciting since this may prevent the development of the HCM phenotype altogether. Developing new treatment options for microvascular ischaemia and studying its beneficial effects on long-term outcomes may support the call for early, aggressive anti-ischaemic treatment in HCM.

## Figures and Tables

**Table 1:** Timeline of events in the early development of HCM (Hypertrophic cardiomyopathy).

1868	First pathological description by Vulpian [6] who reported it as idiopathic hypertrophic subaortic stenosis (IHSS).
1957	Brock [9] reported 3 cases of LVOT hypertrophy and attributed it to systemic hypertension.
1958	Teare [1] published series of 8 autopsy cases who had asymmetrical hypertrophy of the heart, 7 of whom died suddenly. Bercu et al. [10] published a case report on ‘pseudoaortic stenosis’.
1961	Brockenbrough et al. [11] described the Brockenbrough–Braunwald–Morrow sign.
1963	Nonobstructive form of HCM first described [13].
1964	Morrow et al. [19] performed the first surgical myectomy for HCM.
1965	Bjork [20] postulated that SAM caused LVOTO.
1969	Moreyra et al. [21] pioneered the use of M-Mode echocardiography in HCM diagnosis. Shah et al. [22] described SAM in HCM using echocardiography.
1972	Introduction of cross-section/2D echocardiography [23].
1980	First ICD implanted in a patient with HCM [24].
1990	First pathogenic mutation implicated in HCM [25].
1995	Introduction of alcohol septal ablation (ASA) as an alternative to surgical myectomy by Sigwart [26].
2000	First efficacy study on ICD in the prevention of SCD in the HCM population [27].
2002	ACC/AHA/NASPE guidelines [28] recommended (class IIb) the use of ICD in primary prevention of SCD in HCM.

LVOT, Left ventricular outflow tract; SAM, Systolic anterior motion; LVOTO, Left ventricular outflow tract obstruction; ICD, Implantable cardioverter defibrillator; SCD, Sudden cardiac death; ACC/AHA/NASPE, American College of Cardiology/American Heart Association/North American Society for Pacing and Electrophysiology.

**Table 2:** Pathogenic mutations of HCM.

Gene	Protein	Frequency (%)
Cardiac myosin-binding protein C	MYBPC3	30–40%
β cardiac myosin heavy chain	MYH7	20–30%
Cardiac troponin T	TNNT2	5–10%
Cardiac troponin I	TNNI3	4–8%
Regulatory myosin light chain	MYL2	2–4%
Essential myosin light chain	MYL3	1–2%
α tropomyosin	TPM1	<1%
α cardiac actin	ACTC1	<1%
Muscle LIM protein	CSRP3	<1%

## Abbreviations

ACC	American College of Cardiology
ACCF	American College of Cardiology Foundation
AF	Atrial fibrillation
AHA	American Heart Association
ARB	Angiotensin receptor blocker
ASA	Alcohol septal ablation
CMR	Cardiovascular magnetic resonance
DOAC	Direct oral anticoagulant
DT-CMR	Diffusion tensor cardiovascular magnetic resonance
ERA	Endocardial radiofrequency ablation
ESC	European Society of Cardiology
HCM	Hypertrophic cardiomyopathy
ICD	Implantable cardioverter defibrillator
LA	Left atrium
LGE	Late gadolinium enhancement
LV	Left ventricle
LVEF	Left ventricular ejection fraction
LVH	Left ventricular hypertrophy
LVOT	Left ventricular outflow tract
LVOTO	Left ventricular outflow tract obstruction
MV	Mitral valve
NASPE	North American Society of Pacing and Electrophysiology
NYHA	New York Heart Association
PET	Positron Emission Tomography
PICP	C-terminal propeptide of type I procollagen
PVC	Premature ventricular contraction
SAM	Systolic anterior motion
SCD	Sudden cardiac death
VF	Ventricular fibrillation
VT	Ventricular tachycardia
